# Study on Friction and Wear Performance of Sliding Metal Seal Materials Under Reciprocating Motion

**DOI:** 10.3390/ma17205074

**Published:** 2024-10-17

**Authors:** Huiqian Yao, Xiaoyang Liang, Lianchao Guo, Xinpeng Wang, Linqing Bai, Chao Wang

**Affiliations:** 1 Sinopec Research Institute of Petroleum Engineering, Beijing 100101, China; 2 Shelfoil Petroleum Equipment & Services Co., Ltd., Dezhou 253034, China; 3 School of Mechanical & Automotive Engineering, Qingdao University of Technology, Qingdao 266520, China

**Keywords:** sliding seals, metallic materials, surface modification, technology friction and wear

## Abstract

During petroleum drilling, the reciprocating motion in the seal device leads to piston and sleeve wear, which may cause leakage of the sealing medium. Selecting appropriate materials for the piston and sleeve, along with surface modifications, can effectively prolong the seal service life of the seal. The friction and wear properties of piston and sleeve pairs of different materials in a metal sealing device were simulated by the laboratory “pin-on-block” reciprocating friction test. Pins made of 45# steel, 35CrMo, and 20Cr13 were used to simulate piston bulges, while 35CrMo samples were used to simulate sleeves. Additionally, the influence of DLC (diamond-like carbon) coating and QPQ (Quench–Polish–Quench) nitriding on the wear resistance of the materials was studied. Based on this, the friction and wear properties, along with the wear mechanism of different material pairs, were analyzed. The results show that the friction coefficient curves of the three piston base materials and the 35CrMo sleeve are similar, and the friction coefficient of 45# steel is lower than that of 35CrMo and 20Cr13 at the initial stage. The DLC surface coating exhibited the best anti-wear performance, with the lowest friction coefficient, minimal wear, and the most stable friction coefficient. Surface QPQ nitriding treatment can also improve the wear resistance of the base material. However, due to the oxide formed during nitriding being prone to flaking, the friction coefficient fluctuates significantly at the initial stage of testing, and its anti-wear performance was inferior to that of the DLC coating. This study on material pairing and surface modification provides theoretical support for material selection and surface modification design of pistons and sleeves in oil drilling sealing devices.

## 1. Introduction

The wear resistance of the seal material directly impacts the performance and reliability of the seal, which in turn affects the operational efficiency and safety of drilling equipment. As the core components of the sliding sleeve sealing device, the piston and sleeve are subjected to frequent reciprocating friction movement during operation, leading to surface wear and increased risk of leakage.

With the increasing challenges in oil industry mining, higher demands are being placed on the longevity, corrosion resistance, and mechanical properties of sealing devices. Traditional rubber and plastic sealing technologies have struggled to meet these performance requirements, leading to the development of metal sealing technology. Initially utilized for sealing pipe string connections to withstand high pressure and temperature environments, metal sealing technology has evolved to be applied in micro-clearance sealing of oil and gas equipment and tools, such as through the use of metal sealing rings in valves, downhole tools, and pipe joints [1]. Research into the application of metal sealing components to seal units within oil and gas wells with large gaps began in the early 21st century. While progress has been made in China using superelastic metals as packer sealing components, most research remains concentrated in foreign countries [2]. Metal seals demonstrate exceptional durability under extreme conditions like underground high temperatures, pressures, and strong corrosion [3,4]. Compared to elastomer seals, metal-to-metal seals offer advantages, including superior high-temperature stability, higher rated pressure capacity, better chemical compatibility, erosion resistance, and recyclability. These features make metal seals more suitable for applications such as high-temperature/high-pressure oil/gas wells and heavy-oil thermal production [5]. Currently, there is limited research on material pairing surface modification for metal seals. Investigating how material selection surface modification between a metal piston sleeve affects seal device performance is particularly crucial. Surface modification of working materials is necessary to optimize corrosion wear resistance and increase strength. Modified surfaces can exhibit enhanced hardness and improved corrosion wear resistance [6,7,8]. Surface modification technology aims to improve part wear resistance by altering surface structure, depositing coatings, and changing surface composition. In this study, QPQ nitriding treatment and the DLC coating processes were selected variables to investigate their effects on friction wear properties of metallic surfaces.

As an advanced metal surface strengthening technology, QPQ nitriding combines nitriding and oxidation processes to improve the wear resistance and corrosion resistance of metal surfaces through salt bath composite treatment. This treatment technique minimizes workpiece deformation, so it has attracted wide attention in industrial applications [9,10]. It is found that 40Cr steel treated with QPQ has significant advantages in wear resistance compared to conventional quenching, low-carbon carburizing quenching, ion nitriding, hard chromium plating, and other treatments. The wear resistance of a 5Cr21Mn9Ni4N steel valve treated with QPQ is approximately twice as high as that of a hard chrome-plated valve [11,12]. The research of Guo Jie et al. [13] indicates that the friction coefficient and wear amount of QPQ-treated samples are reduced to varying degrees under dry friction, oil lubrication, and impact loads. Alberto et al. [14] found that QPQ technology can improve the wear resistance and corrosion resistance of materials, which is achieved by increasing the surface hardness and residual compressive stress of materials. The study of Khan et al. [15] showed that, compared with MoS2 coating, QPQ treatment can significantly improve the wear resistance of steel substrate. Additionally, this technology is compatible with a wide range of materials, has low production costs, and offers broad application prospects [16].

Diamond-like carbon (DLC), as a hard self-lubricating film material, exhibits exceptional mechanical and tribological properties, including high hardness, high elastic modulus, low friction, and superior wear resistance [17,18,19]. It has been widely applied across various engineering fields [20,21,22,23], including ceramic materials [24,25], metal materials [26,27], and soft materials [28]. Research indicates that the DLC coating can significantly enhance the surface hardness and corrosion resistance of alloy substrates. Seyed Elias et al. [29] found that the DLC coating effectively increases the surface hardness of alloy matrices and reduces their friction coefficient from 0.2 to 0.13. Furthermore, Mannan et al.’s [30] research findings demonstrate that under oil lubrication conditions, among two paired auxiliary materials of steel and DLC, the DLC/DLC combination exhibits the smallest friction coefficient, followed by steel/DLC, while steel/steel shows the largest value. This indicates that the DLC coating can effectively reduce friction coefficients.

Based on our newly developed metal sliding seal, which has been proven to have a good sealing effect, this study aims to provide a more reliable and efficient material selection scheme for oil drilling tools by comparing the pairing relationship between piston and sleeve materials and two different surface modification methods, DLC coating and QPQ nitriding treatment. This study will provide a more reliable and efficient material selection scheme for oil drilling tools and seeks to extend the reliability and service life of the equipment. Additionally, it will offer theoretical support for the design and optimization of oil drilling seals.

## 2. Experimental Details

### 2.1. Experimental Equipment

**Figure 1:**
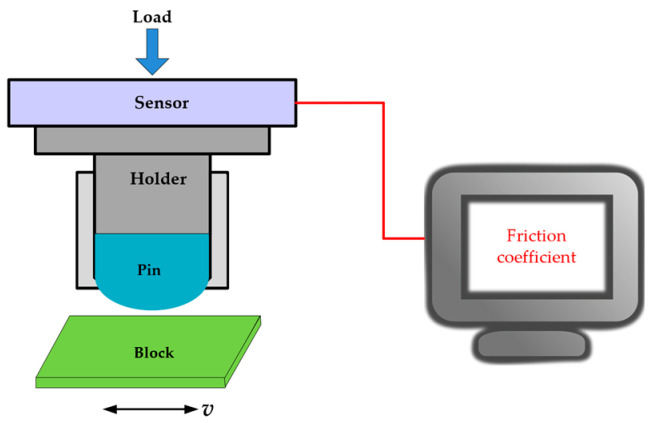
Schematic diagram of the UMT tester.

The test was carried out on the UMT friction and wear testing machine as shown in Figure 1. The upper pin serves to simulate the piston bulge, while the lower block is used to simulate the sleeve. The pin is fixed in a special fixture and remains stationary during the experiment, while the lower block moves reciprocally at the set frequency. The applied load was determined based on the equivalent of the calculated contact stress.

After the reciprocating wear test, the samples were cleaned ultrasonically using alcohol and deionized water. A white light surface topography instrument was employed to capture the three-dimensional morphology of the worn surface of the specimen and measure the wear volume. The wear morphology and elemental composition were subsequently analyzed using scanning electron microscopy (SEM, ZEISS, Oberkochen, Germany).

### 2.2. Test Materials and Preparation

During the test, pistons made from 45#, 35CrMo, and 20Cr13, along with DLC-coated variants (45#-DLC, 35CrMo-DLC, and 20Cr13-DLC) and QPQ-treated variants (45#-QPQ, 35CrMo-QPQ, and 20Cr13-QPQ), were utilized in conjunction with a sleeve made from 35CrMo. The basic parameters of the test materials at room temperature are summarized in Table 1. Reciprocating wear tests were conducted between the pins and blocks to simulate the operational conditions of pistons and sleeves in practical applications. The initial surface roughness of pins and the sample block were measured as Ra = 50 nm and Ra = 150 nm, respectively.

**Figure 2:**
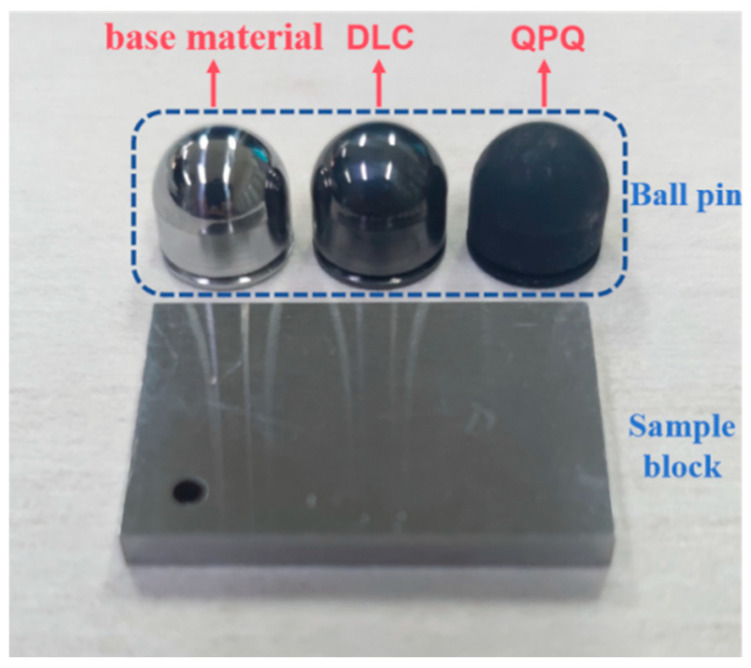
Samples diagram.

The DLC coating utilized in this research was prepared using the plasma-assisted chemical vapor deposition (PACVD) process. First, the deposition chamber pressure was evacuated to 2.2 × 10^−3^ mbar, and the test block was cleaned with Ar plasma for 15 min at 8.2 × 10^−2^ mbar pressure and 500V voltage. Under the working conditions of 4 × 10^−1^ mbar working pressure, 550 V deposition voltage, and an Ar:CH4 ratio of 3:1, the duty cycle was adjusted to 40%, the frequency was 20 kHz, the deposition temperature was about 145 °C, and the DLC sample was obtained after continuous deposition for 30 min. QPQ treatment was to keep the sample block in a salt nitriding bath at 570 °C for 120 min, and then keep it in a salt oxide bath at 400 °C for 60 min. The specific process consisted of preheating, salt bath nitriding–polishing, secondary oxidation, cleaning, and drying to obtain the QPQ sample block [31]. The sample diagram is presented in Figure 2.

### 2.3. Experimental Methods

The Hertzian contact pressure of the sealing pair under actual working conditions corresponds to a load of 10 N in the pin-on-block contact test. The reciprocating friction experiments were carried out under dry friction conditions at room temperature with a load of 10 N, a frequency of 2 Hz, and a reciprocating stroke of 6 mm. As wear of the friction pair primarily occurs in the initial stage, the test duration was set to 3 min to simulate this phase. To ensure the reliability of the experimental data, each set of experiments was repeated twice.

## 3. Results and Discussions

### 3.1. Friction Coefficient Analysis

**Figure 3:**
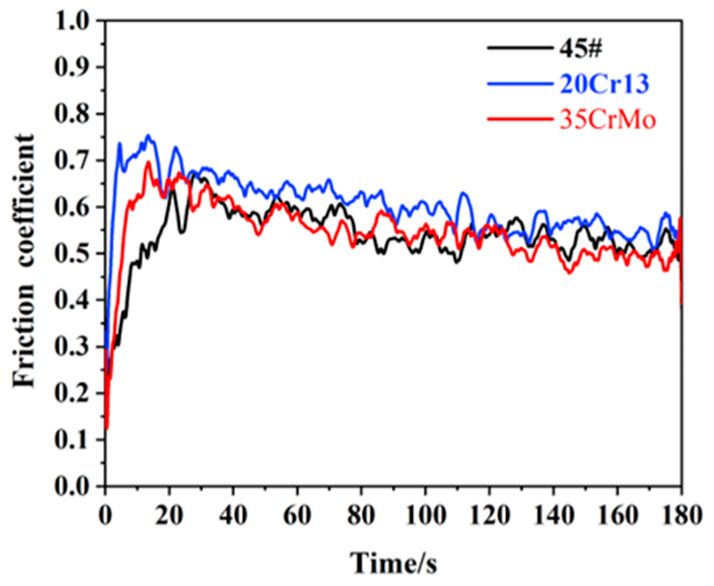
Friction coefficient of basic material.

Figure 3 presents the variation in the friction coefficient over time for pins made from 45#, 20Cr13, and 35CrMo, paired with a 35CrMo sample block. As can be seen from the figure, when unmodified pins are tested against the 35CrMo sample, the friction coefficient of any material will fluctuate greatly. The average friction coefficient in the early stage increases rapidly, with 20Cr13 showing the fastest rise, followed by 35CrMo. In contrast, 45# exhibits a delayed peak after the initial rapid rise of the other two materials. With the increase in wear time, the friction coefficient gradually decreases and stabilizes, of which 20Cr13 has the largest, maintaining about 0.6. 20Cr13 and 45# are relatively similar, maintaining around 0.55. The main reason for this phenomenon is the existence of roughness peaks on the contact surfaces of friction pairs. When friction begins, the pressure at these roughness peaks is high, leading to intense wear. With the increase in friction time, wear continued, the appearance of the contact surface of the friction pair was gradually improved, and the rough peak became smoothed or adjusted, increasing the actual contact area and making it more uniform. This makes the coefficient of friction gradually stabilize, because with the improvement of the contact surface, the change of peak pressure decreases, thus slowing the rate of change of the coefficient of friction [32].

**Figure 4:**
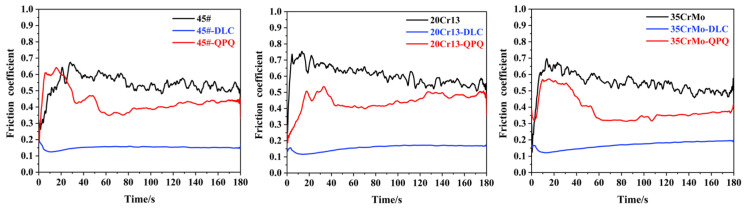
Influence of surface modification on the friction coefficient.

Figure 4 shows the friction coefficient of the 35CrMo sample block with 45#, 20Cr13, and 35CrMo pins and corresponding DLC coating pins (45#-DLC, 20CR13-DLC, 35CRMO-DLC) and QPQ nitriding treatment pins (45#-QPQ, 20CR13-QPQ). It is evident that the friction coefficient of the three base material pins during the reciprocating process was higher than those of the base material after DLC coating and QPQ nitriding treatment. This indicates that both DLC coating and QPQ nitriding significantly improve the frictional properties of the base material. Among them, when 45#-QPQ, 20Cr13-QPQ, 35CRMO-QPQ, and 35CrMo are sliding, the friction coefficient is high at the beginning, and then there is a significant decline. This behavior is attributed to the oxide layer formed on the surface after QPQ treatment. In the early stage of the reciprocating friction process, the uneven oxide falls off, resulting in large friction coefficient values and fluctuations. As friction progresses, the detached oxide particles act as a lubricant within the contact zone, causing a sharp reduction in the friction coefficient. Eventually, the nitrided base material participates in the wear process, leading to a gradual stabilization of the friction coefficient. In contrast, when the pins are DLC-coated (45#-DLC, 20Cr13-DLC, 35CrMo-DLC), the friction coefficient is the lowest and most stable. With the progress of friction, the friction coefficient shows a slow rising trend, which is because the outermost DLC layer contains a high proportion of sp2-C. This layer is prone to graphitization, which lubricates the friction process by reducing wear and maintaining low friction levels [33].

Based on the analysis of the friction behavior for different pin materials and their corresponding surface modifications on the friction coefficient as depicted in Figure 3 and Figure 4, it is clear that the friction coefficient of 45# steel, 45#-DLC, and 35CrMo-DLC pins against the 35CrMo sample block is the lowest. From the standpoint of optimizing frictional properties, 45# steel and its modified versions should be prioritized. Subsequent tests on the friction surface morphology primarily focused on the friction pairs composed of 45# steel, 45#-DLC, and 35CRMO-DLC pins with 35CrMo samples. The wear characteristics and underlying wear mechanisms were further analyzed through detailed examination of the wear morphology.

### 3.2. Wear Topography Analysis

**Figure 5:**
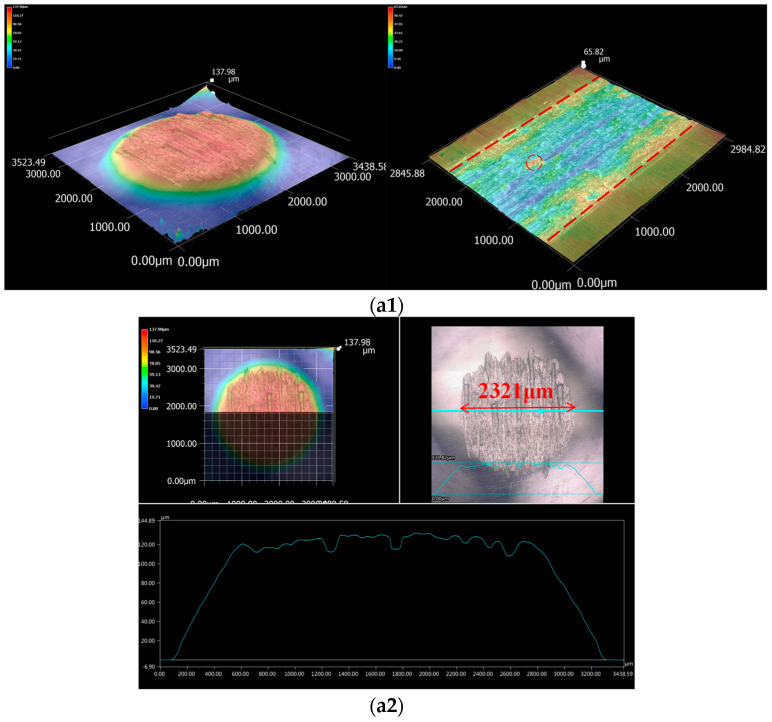

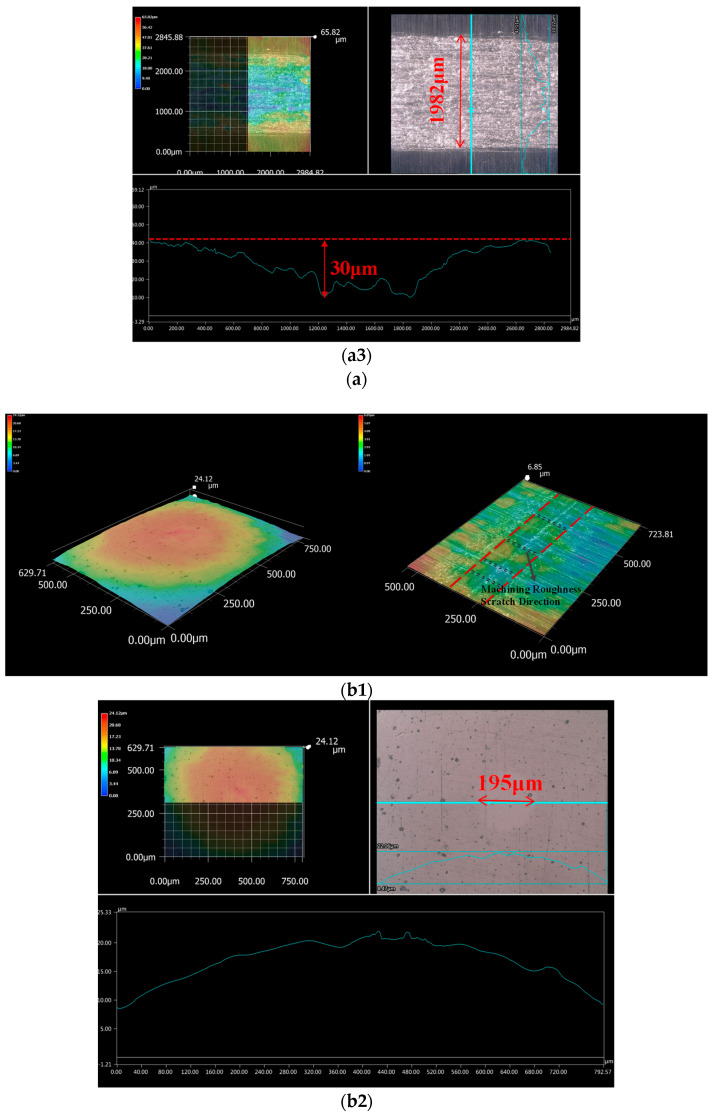

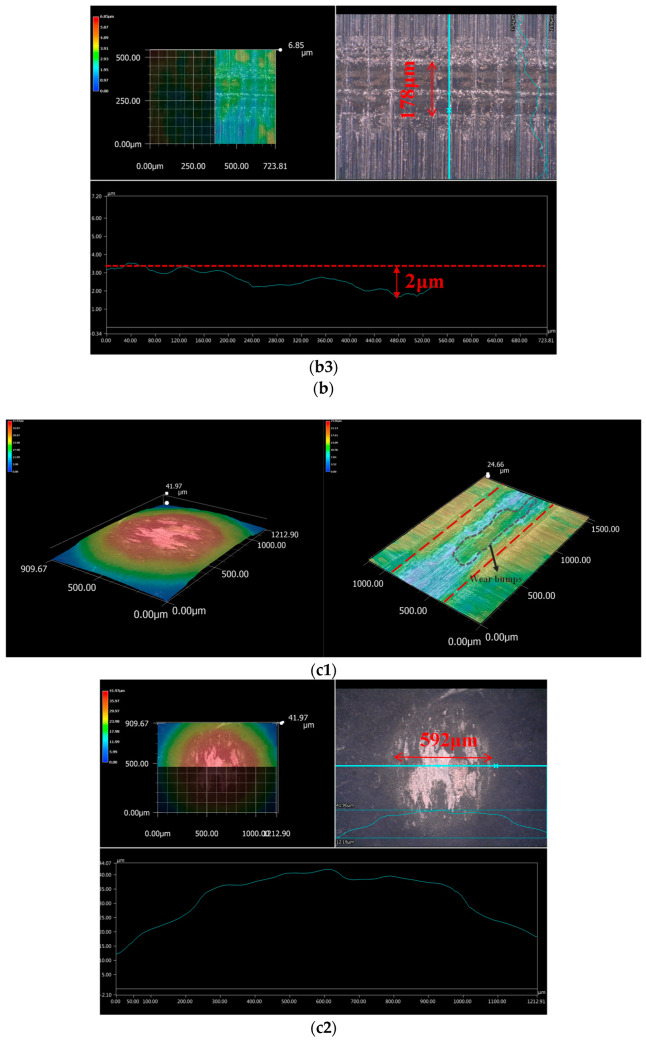

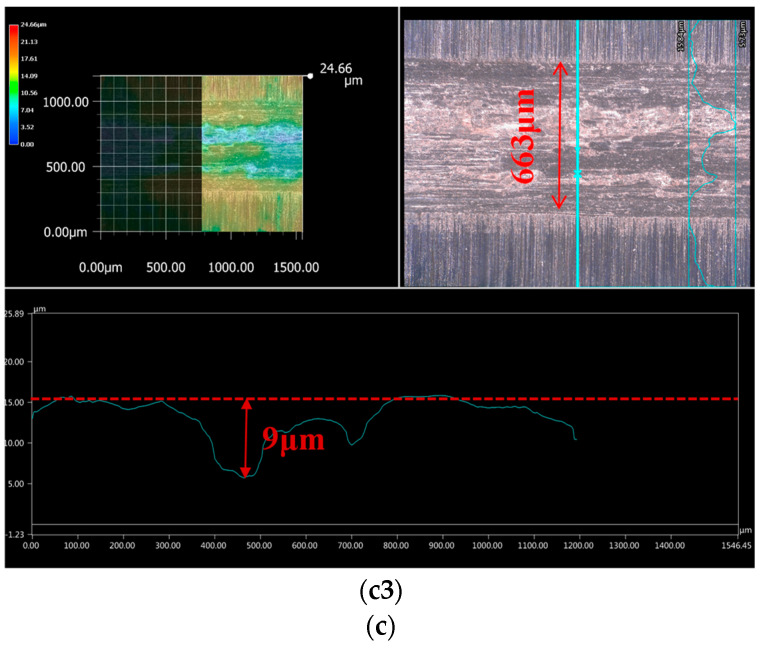
White light topography: (**a**) 45# pin—35CrMo block; (**a1**) 3D wear scar of 45# pin and 35CrMo block; (**a2**) 2D wear scar of 45# pin; (**a3**) 2D wear scar of 35CrMo block; (**b**) 45#-DLC pin—35CrMo block; (**b1**) 3D wear scar of 45#-DLC pin and 35CrMo block; (**b2**) 2D wear scar of 45#-DLC pin; (**b3**) 2D wear scar of 35CrMo block; (**c**) 45#-QPQ pin—35CrMo block; (**c1**) 3D wear scar of 45#-QPQ pin and 35CrMo block; (**c2**) 2D wear scar of 45#-QPQ pin; (**c3**) 2D wear scar of 35CrMo block.

Figure 5 shows the white light 3D topography of the contact area between the pins of 45# steel, 45#-DLC, 45#-QPQ, and 35CrMo samples after the reciprocating friction and wear test. The top part of each diagram is the wear morphology of the pins, and the bottom part is the wear morphology of the block. As observed, the surface wear of the 45# steel pin and 35CrMo block is the most serious when they are directly grinding. Obvious furrows can be seen in the contact area between the pin and sample. The surface of the pin was worn to a diameter of approximately 2321 μm, while the width and depth of the 35CrMo block were about 2000 μm and 30 μm, respectively. When the 45#-DLC pin was paired with the 35CrMo block, only slight wear spots were observed on the pin surface, with no evident detachment of the DLC coating. The original grinding marks were visible in the wear zone of the 35CrMo block, indicating that the wear amount in the contact zone of the friction pair was small. As can be seen from Figure 5b, there are only abrasion spots with a diameter of about 195 μm on the surface of the pin; the width and depth of the abrasion marks on the surface of the block are about 178 μm and 2 μm, respectively. During the grinding process of the 45#-QPQ pin and the 35CrMo block, the oxide peel falls off into the contact zone at the initial wear stage due to the uneven oxide on the surface of the 45#-QPQ pin, which leads to the base material being exposed on the pin surface as shown in Figure 5c. At the end of the wear, a wear spot with a diameter of about 592 μm was formed on the surface of the pin, and the width of the 35CrMo sample was about 200 μm and the depth was about 9 μm. According to the three-dimensional wear morphology analysis in the contact area, when the sample material is 35CrMo, the surface modification methods of the two materials selected in this study can both improve the wear resistance, and the best wear resistance of the pin material is 45#-DLC, followed by 45#-QPQ, and the worst is 45# steel.

### 3.3. Wear Characteristics

**Figure 6:**
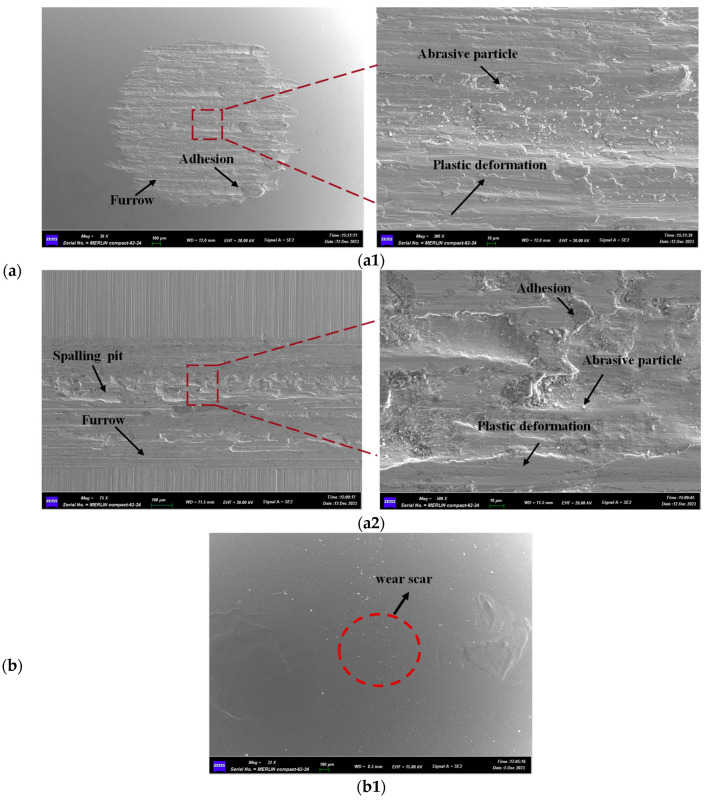

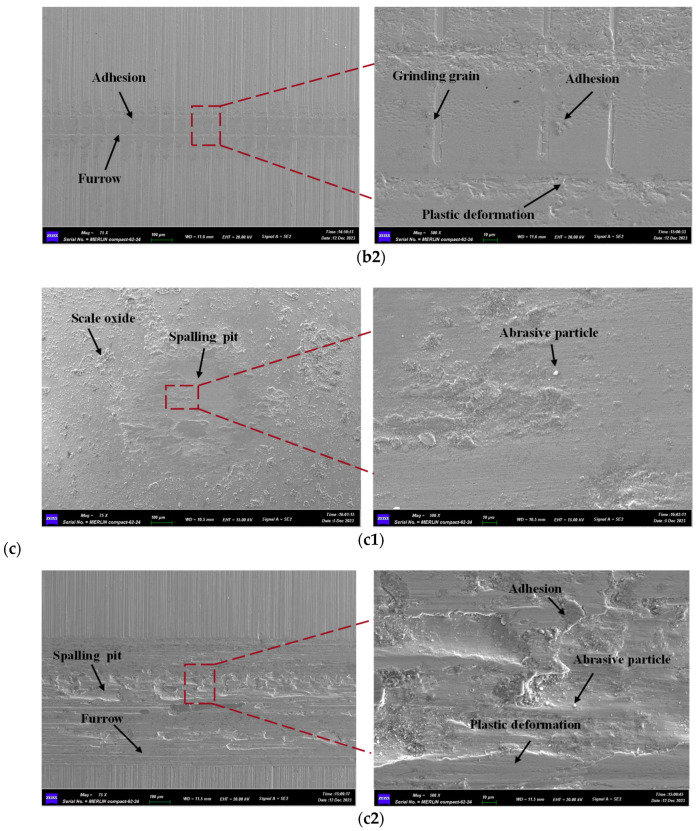
SEM image of wear scar: (**a**) 45# pin—35CrMo block; (**a1**) 45# pin; (**a2**) 35CrMo block; (**b**) 45#-DLC pin—35CrMo block; (**b1**) 45#-DLC pin; (**b2**) 35CrMo block; (**c**) 45#-QPQ pin—35CrMo block; (**c1**) 45#-QPQ pin; (**c2**) 35CrMo block.

Figure 6 presents the SEM images of the wear zones on the 45# steel, 45#-DLC, and 45#-QPQ pins after undergoing reciprocating friction against the 35CrMo sample. As shown in Figure 6(a1), the wear on the 45# steel pin and the 35CrMo sample is relatively severe, with the wear area primarily characterized by furrow and adhesive wear, along with localized plastic deformation. Upon magnification, wear particles can also be observed. At the onset of reciprocating friction, the contact pressure between the 45# steel pin and the rough peaks of the 35CrMo sample block is significant, causing the material at the rough peaks to melt and shear, leading to adhesive wear. The partially sheared rough peaks and detached adhesive particles form abrasive debris between the contact surfaces, contributing to the wear process. This results in repeated adhesion and spalling of the abrasive particles, eventually leading to the formation of adhesive pits. As sliding progresses, some regions undergo rolling and plastic deformation.

Since a clear image of the wear zone on the 45#-DLC pin could not be captured using electron microscopy after wear (Figure 6(b1)), the microscopic analysis focuses solely on the wear zone of the corresponding 35CrMo sample. As seen in Figure 6(b2), after friction with the 45#-DLC pin, distinct grinding marks remain visible in the wear zone on the surface of the 35CrMo sample block, indicating minimal surface wear. The wear observed is primarily characterized by furrows, with minor adhesive wear and plastic deformation. According to the three-dimensional white light topography shown in Figure 5b, the 45#-DLC pin experienced slight wear. The formation of furrows is likely due to the transfer of the worn DLC film into the contact zone. Given the high hardness of the DLC film, these furrows are generated as the DLC particles scrape against the softer surface of the 35CrMo sample block during sliding. The minor adhesive wear is caused by material from the sample being scraped off and adhering to the pin surface. However, due to the excellent lubricating properties of the DLC coating, the overall wear on the sample remains minimal.

Figure 6c presents the SEM image of the wear zone between the 45#-QPQ pin and the 35CrMo sample. In Figure 6(c1), the uneven patches outside the wear zone represent the oxide layer formed during the QPQ nitriding process on the pin surface. This oxide layer exhibits poor adhesion to the substrate and tends to detach during friction. The smooth region within the wear zone reveals the exposed base material after wear. The enlarged view of the wear mark shows that remnants of the oxide layer still adhere to the wear zone after the friction test, indicating that the QPQ treatment improves the material’s wear resistance. In the case of the 35CrMo sample, significant adhesive wear is evident during the sliding process, with numerous adhesive pits scattered across the contact area. This occurs because the increased hardness and wear resistance of the pin make the 35CrMo sample act as a sacrificial material during friction, leading to the formation of adhesion pits predominantly on the sample surface.

An analysis of the friction coefficient and wear morphology reveals that both DLC coating and QPQ nitriding treatment effectively enhance the wear resistance of pins and sample block friction pairs, and the DLC coating demonstrates a more significant impact in reducing wear and improving anti-wear performance. This is attributed to the fact that, although both the DLC film and QPQ oxide film provide lubricating effects, the harder DLC film exhibits stronger adhesion, whereas the QPQ oxide layer tends to wear off more easily. Once the oxide layer wears away, adhesive wear predominantly occurs on the surface of the sample due to the increased hardness of the nitrided base material. Therefore, from the perspective of enhancing wear resistance, DLC coating is recommended as the preferred method for enhancing the sealing effect.

## 4. Conclusions

The wear resistance of the material is an important factor affecting the sealing performance of metal sliding seals. This study simulates the reciprocating sliding process of the piston protrusion and sleeve in a metal sliding seal using a pin-sample reciprocating wear test. The optimal matching performance of 45# steel, 20Cr13, and 35CrMo piston materials with a 35CrMo sleeve was analyzed. Based on this, the influence of DLC coating and QPQ nitriding on wear resistance was examined. The following conclusions are obtained:

(1) The final friction coefficient of the piston materials of 45# steel, 20Cr13, and sleeve piston materials of 35CrMo is not significantly different. However, the initial friction coefficient of 45# steel is lower, making it the preferred choice for the piston material.
(2) The sliding friction coefficient of the pin after surface QPQ treatment can be significantly reduced, but the coefficient exhibits considerable fluctuations due to the peeling of the oxide layer from the surface of the QPQ-treated material during the initial sliding phase. The most significant anti-wear and anti-friction effect is obtained by coating the surface of the pins with the DLC film. The lowest friction coefficient and the smallest wear rate are obtained, and the coefficient fluctuates the least.
(3) The optimal material pairing for reducing the friction coefficient is identified as 45# steel for the piston and 35CrMo for the sleeve, while the most effective surface treatment for enhancing wear resistance is the DLC coating, followed by QPQ treatment.

## Figures and Tables

**Table 1:** Test material parameters at ordinary temperature.

Materials	Modulus of Elasticity	Yield Strength	Poisson’s Ratio	Tangent Modulus
45#	231 GPa	448 MPa	0.3	4000 MPa
20Cr13	222 GPa	880 MPa	0.3	4223 MPa
35CrMo	208 GPa	832 MPa	0.3	3064 MPa

## Data Availability

The original contributions presented in the study are included in the article, further inquiries can be directed to the corresponding author.
